# Complete Genome Sequence of a *Salivirus* in Respiratory Specimens from a Child with Adenovirus Infection

**DOI:** 10.1128/genomeA.00159-16

**Published:** 2016-04-07

**Authors:** Na Pei, Jinmin Ma, Liqiang Li, Jingkai Ji, Jiandong Li, Jikui Deng, Xin Liu

**Affiliations:** a BGI-Shenzhen, Shenzhen, China; b Shenzhen Children's Hospital, Shenzhen, China

## Abstract

*Salivirus* is a new member of the family *Picornaviridae* and is associated with diarrhea, especially in children, being often found in feces. Here, we report the complete genome sequence of a *Salivirus* strain in respiratory specimens from a child with adenovirus infection.

## GENOME ANNOUNCEMENT

*Salivirus* was documented as being associated with diarrhea in some previous studies (1, 2). *Salivirus* was detected in stool samples from Nigeria, Tunisia, Nepal, and the United States in 2009 (3). Viruses with approximately 90% nucleotide similarity, named klassevirus, were also reported in three cases of unexplained diarrhea from the United States and Australia and in sewage from Spain (4–7). Until 2010, *Salivirus* in fecal samples from children with diarrhea in China was newly characterized in Shanghai (8). To our knowledge, there have been no reports of infection with *Salivirus/Klassevirus* in respiratory samples, and the effect of klassevirus on humans remains unclear. Han et al. (9) analyzed 142 nasopharyngeal samples in 2010 but did not detect any *Salivirus/Klassevirus*.

A 7-month-old boy was admitted to Shenzhen Children’s Hospital because of adenovirus infection. One nasopharyngeal swab was collected from this fatal child, and the presence of the *Salivirus* in the RNA sample was found using a metagenomics method. This was the first *Salivirus* case found to be associated with respiratory infection. Therefore, the complete genome of this new isolate, *Salivirus* A SZ1, was sequenced by next-generation sequencing. Total RNA was extracted from the sample using Qiagen RNeasy kit (Qiagen, Inc., Germany). The sequencing library was prepared using the RNA library preparation kit (Life, Inc., NY). Sequencing was performed on an Ion Proton platform (Life, Inc.) for 200 cycles (single-end). Raw reads were trimmed for high quality and assembled using the IDBA-ud (1.1.1). Virus contigs were obtained, and specific primers were designed to fill the gaps using Sanger sequencing.

*Salivirus* A SZ1 contains a genome with a size of 7,633 nucleotides (nt) encoding a polyprotein of 2,331 amino acids (aa), excluding the 3′ tail. The coding sequence (CDS) was located between nt 651 and nt 7633. Polyprotein genes were arranged as follows: L gene, nt 651 to 989; VP0 gene, nt 990 to 2090; VP3 gene, nt 2091 to 2762; VP1 gene, nt 2763 to 3590; 2A, nt 3591 to 3947; 2B, nt 3948 to 4409; 2C, nt 4410 to 5447; 3A, nt 5448 to 5678; 3B, nt 5679 to 5765; 3C, nt 5766 to 6350; and 3D, nt 6351 to 7633.

*Salivirus* A SZ1 was most closely related to *Salivirus* A isolate BN-2 in Germany (97% nucleotide similarity) at the whole-genome level and the *Salivirus* A strain in Shanghai (accession no. GU245894) (96%) isolated in 2010. *Salivirus* A SZ1 was less closely related to the *Salivirus* A isolate 02394-01.

In conclusion, a *Salivirus* strain in respiratory specimens from a child with adenovirus infection was characterized. Our finding of *Salivirus* in children is the first case, to our knowledge, in respiratory samples. Continued study and more investigation of the virus are needed.

### Nucleotide sequence accession number.

The complete genome sequence of *Salivirus* A SZ1 has been deposited at GenBank under the accession no. KT182636.
